# Health effects of home energy efficiency interventions in England: a modelling study

**DOI:** 10.1136/bmjopen-2014-007298

**Published:** 2015-04-27

**Authors:** Ian Hamilton, James Milner, Zaid Chalabi, Payel Das, Benjamin Jones, Clive Shrubsole, Mike Davies, Paul Wilkinson

**Affiliations:** 1 UCL Energy Institute, University College London, London, UK; 2 Department of Social & Environmental Health Research, London School of Hygiene & Tropical Medicine, London, UK; 3 UCL Institute for Environmental Design and Engineering, University College London, London, UK; 4 Department of Architecture and Built Environment, University of Nottingham, Nottingham, UK

**Keywords:** EPIDEMIOLOGY, PUBLIC HEALTH

## Abstract

**Objective:**

To assess potential public health impacts of changes to indoor air quality and temperature due to energy efficiency retrofits in English dwellings to meet 2030 carbon reduction targets.

**Design:**

Health impact modelling study.

**Setting:**

England.

**Participants:**

English household population.

**Intervention:**

Three retrofit scenarios were modelled: (1) fabric and ventilation retrofits installed assuming building regulations are met; (2) as with scenario (1) but with additional ventilation for homes at risk of poor ventilation; (3) as with scenario (1) but with no additional ventilation to illustrate the potential risk of weak regulations and non-compliance.

**Main outcome:**

Primary outcomes were changes in quality adjusted life years (QALYs) over 50 years from cardiorespiratory diseases, lung cancer, asthma and common mental disorders due to changes in indoor air pollutants, including secondhand tobacco smoke, PM_2.5_ from indoor and outdoor sources, radon, mould, and indoor winter temperatures.

**Results:**

The modelling study estimates showed that scenario (1) resulted in positive effects on net mortality and morbidity of 2241 (95% credible intervals (CI) 2085 to 2397) QALYs per 10 000 persons over 50 years follow-up due to improved temperatures and reduced exposure to indoor pollutants, despite an increase in exposure to outdoor-generated particulate matter with a diameter of 2.5 μm or less (PM_2.5_). Scenario (2) resulted in a negative impact of −728 (95% CI −864 to −592) QALYs per 10 000 persons over 50 years due to an overall increase in indoor pollutant exposures. Scenario (3) resulted in −539 (95% CI −678 to -399) QALYs per 10 000 persons over 50 years follow-up due to an increase in indoor exposures despite the targeting of pollutants.

**Conclusions:**

If properly implemented alongside ventilation, energy efficiency retrofits in housing can improve health by reducing exposure to cold and air pollutants. Maximising the health benefits requires careful understanding of the balance of changes in pollutant exposures, highlighting the importance of ventilation to mitigate the risk of poor indoor air quality.

**Strengths and limitations of this study**

- The epidemiological evidence about health effects associated with indoor air pollutants and thermal stress is of varying certainty, though more evidence exists for exposure to outdoor pollution and temperature; therefore, only exposures with strong evidence were used.
- This study uses advanced validated building physics models to determine the change in indoor pollutant and thermal exposures related to energy efficiency retrofits.
- The uncertainty in the exposure responses on estimates of health impacts, such as the estimates for cold-related deaths, the toxicity level of particles derived from indoor sources and mental health, could result in a different balance of pollution impact depending on the assumptions made.
- While offering policymakers a support tool to include health as a criterion when developing and assessing home energy efficiency policy, the results presented here should be viewed with a clear understanding of the limitations associated with a modelling study.

## Introduction

By 2030, the UK housing stock will undergo major changes to improve its energy performance,^1^ motivated by the need to reduce emissions of greenhouse gases (GHGs), considerations of energy security/cost, and concern about fuel poverty with its presumed link to the UK's large burden of winter/cold-related mortality and morbidity.^2^ Housing is responsible for one-quarter of total UK CO_2_ emissions^3^ and 52% of this is from space heating. Meeting the UK's ambitious energy efficiency targets will require investments to upgrade the energy performance of nearly all dwellings by 2030.^1^ These changes to housing energy performance will comprise one of the largest natural experiments in the indoor environment in the coming decades and these are likely to have major impacts on the indoor environment and population health.^4^ ^5^ To date, health consequences have received limited examination,^6^ though they are increasingly being recognised as an issue by the UK Government.^7^

**Figure 1:**
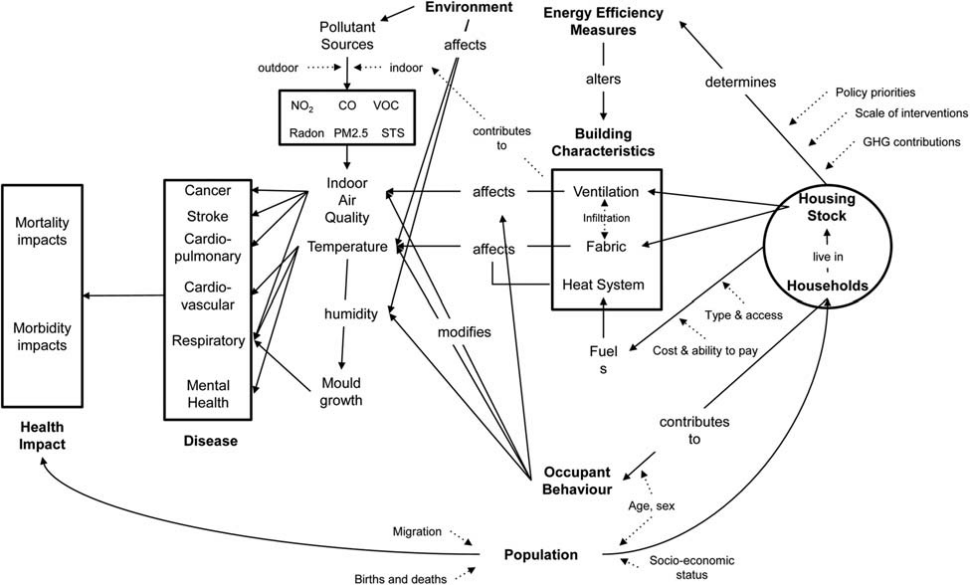
Connections between energy efficiency in housing and health (GHG, greenhouse gas; STS, secondhand tobacco smoke; VOC, volatile organic compound).

Properly designed and implemented, actions to improve housing energy performance could have major co-benefits for public health,^4^ although there are risks involved and the possibility of poorly designed interventions leading to unintended consequences (figure 1).^8–10^ Energy efficiency retrofits that alter the fabric heat loss can also increase the air tightness of the dwelling,^11^ ^12^ increasing exposure to indoor-generated pollutants (eg, particulates, mould, radon). Living in cold or inefficient and poorly ventilated homes is linked to a range of health problems.^5^ ^10^ ^13^ Retrofits that improve indoor temperatures may have positive impacts on mental health and cardiorespiratory disease,^5^ but could have negative impacts on respiratory conditions due to the increased levels of indoor pollutants.^14^ ^15^ In the UK, most of our time is spent indoors and the majority of the health impact of more airtight buildings is likely to occur over the long term through low-dose exposure.^16^

While current English building regulations requires that adequate means of ventilation is provided to dwellings,^17^ there is a lack of guidance for determining the level of ventilation required to protect health before or following an energy efficiency retrofit.^18^ The only guidance that exists relates to the replacement of existing window trickle vents. Ultimately, additional ventilation following a retrofit is left to the discretion of the installer or household. The aim of this study is to illustrate the potential health impact of energy efficiency retrofits under different ventilation settings.

In this paper, we describe the results of a modelling study to quantify changes in exposures in the indoor environment and their associated health consequences attributable to housing energy efficiency retrofits. We do this to characterise possible health-related consequences in need of further scrutiny for the development of national policies and guidance on housing energy efficiency interventions. By doing so, we attempt to gain a better understanding of the trade-offs between risks and benefits for population health.

## Methods

We developed a household-level model to quantify the principal exposure and health pathways outlined in figure 1. The model comprised two parts:

1. A building physics model of English houses that quantifies indoor winter temperatures, exposures to particle pollution, secondhand tobacco smoke (STS), radon, mould growth and energy demand in relation to the energy performance of the dwelling; and
2. A model of the resulting health impacts based on a combination of life table methods and directly modelled changes in disease prevalence.

**Figure 2:**
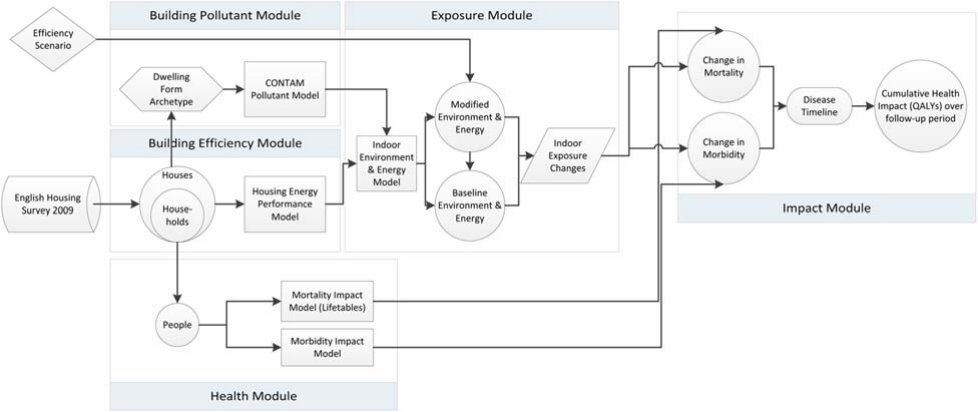
Health Impact of Domestic Energy Efficiency Model (HIDEEM) conceptual framework. The figure demonstrates the components of the model with solid lines representing input flows.

The two model components make up the Health Impact of Domestic Energy Efficiency Model (HIDEEM; figure 2), an exposure-determinant and health impact model.

Other health outcomes that could be related to energy efficiency interventions but were not considered here include cold-related falls, changes in mental health impact (aside from temperature) and some forms of indoor pollutants (eg, volatile organic compounds, carbon monoxide poisoning, dust mites). However, such evidence can be sparse and the exposure–response uncertain. We have not modelled the impact of cold on respiratory disease (eg, chronic obstructive pulmonary disease) because the evidence required for robust quantification is still equivocal;^19^ we hope to address this in future versions of the model. Also, we have not modelled the risk of overheating on energy efficiency, though this could have an important impact in the future. A difficulty with many empirical studies looking at the health effect of energy efficiency interventions is that the study designs and methods have not been sufficiently robust in their design or controlling for bias so as to draw strong conclusions.^5^

### 

#### Part 1: Modelling the indoor environment

We developed a model that characterised the indoor environmental conditions of the 2010 English Housing Survey (EHS).^20^ The indoor environmental conditions and changes in those conditions related to energy efficiency interventions were modelled using validated building physics and airflow models.^21–23^ The modelling, described in detail elsewhere,^16^ ^24^ ^25^ used representative archetype dwelling forms (informed by sampling from the EHS^26^ ^27^) to represent the English dwelling stock. Each of these archetypes was modelled under different levels of air tightness and ventilation systems: window opening only, window trickle vents, extract fans, and combined use of trickle vents and extract fans. A total of 896 archetypes were modelled and matched to the EHS on the basis of dwelling type (eg, detached, semidetached, terraces and flats), floor area and notional permeability. The result was a model of indoor environmental conditions for a representative sample of English dwellings (see online supplementary appendix 1 for further details).

Dwelling energy performance was calculated as a notional heat loss value.^12^ We used an empirical relationship between the dwelling heat loss value and standardised internal temperature (SIT)^i^ to predict the bedroom and living room temperature, standardised at an external temperature of 5°C.^12^ ^28^ The SIT is a measure of the thermal condition of the dwelling ranked against all other dwellings, and is a function of the dwelling’s energy and ventilation performance. The estimated average SIT (derived from an average temperature of the living room and bedroom) for each dwelling reflects the observed distribution shown in Oreszczyn *et al*.^11^ The SIT to thermal performance relationship used in the model captures empirical rebound in temperature (eg, reduced heat flow, changes in occupant heating practices and temperature increases).^12^ We used EHS data on dwelling fabric characteristics, heating system type and presence of ventilation systems to determine eligibility for energy efficiency upgrades (see online supplementary appendix 2).

#### Part 2: Quantification of health impact

We focused on a relatively restricted list of exposures that are supported by reasonably clear epidemiological evidence.^5^ The health impact of changes in indoor air quality and temperature on (cause-specific) mortality was modelled using life table methods based on the IOMLIFET model^29^ but applied to individuals in the EHS data based on their age, sex and specific exposure changes. Life tables were set up using 2010 age-specific population and (disease-specific and all-cause) mortality data for England and Wales from the Office for National Statistics (ONS), with separate life tables set up for males and females.^30^ We modelled changes in five indoor exposures: SIT, STS, indoor and outdoor sources of particulate matter with a diameter of 2.5 μm or less (PM_2.5_), radon and mould; the selected outcomes are listed in table 1. Impacts on morbidity for these same outcomes were estimated from the mortality estimates by applying age-specific and cause-specific ratios of years of healthy life lost due to disability (YLD) to the overall years of life lost (YLL) derived from WHO Global Burden of Disease data.^31^

**Table 1:** Mortality and morbidity outcomes modelled and exposure–response relationships

Exposure	Health outcome	Exposure–response relationship	Reference
Mortality			
Standardised internal temperature	Winter excess cardiovascular (including excess cerebrovascular accident and myocardial infarction)	0.98 per °C	Derived from ref. 32
Secondhand tobacco smoke	Cerebrovascular accident	1.25 (if in same dwelling as smoker)	33
	Myocardial infarction	1.30 (if in same dwelling as smoker)	34
PM_2.5_	Cardiopulmonary	1.082 per 10 µg/m^3^	35 36
	Lung cancer	1.059 per 10 µg/m^3^	As above
Radon	Lung cancer	1.16 per 100 Bq/m^3^	37
Morbidity			
Standardised internal temperature (°C)	Mental health: Common mental disorders (GHQ-12 score 4+)	0.90 per °C	Based on Warm Front^38^
Mould (% MSI >1)	Asthma		
	Harm class II (hospital admission)	1.53 per 100%	Based on ref. 39 and used in HHSRS*
	Harm class III (GP consultation)	1.53 per 100%	As above
	Harm class IV (minor symptoms)	1.83 per 100%	As above

*Housing health and safety rating system.

GHQ, General Health Questionnaire; GP, general practitioner; HHSRS, housing health and safety rating system; MSI, mould severity index; PM_2.5_, particulate matter with a diameter of 2.5 μm or less.

Since some of the outcomes are subcategories of others, to avoid double counting we removed deaths in those subcategories from the larger categories. For outcomes affected by more than one exposure, we assumed the relative risks were multiplicative.

We assumed no time lags for cold-related deaths since these would likely to begin to occur within a year. For the other outcomes, a change in exposure would not necessarily lead to an immediate change in mortality in the population. Therefore, we incorporated disease-specific time functions to account for disease onset and cessation lags over time. The time lag functions were based on empirical evidence of the effect of smoking cessation on mortality over time,^40^ and plausible assumptions about disease progression over time (see online supplementary appendix 3).

We separately estimated morbidity impacts on common mental disorders (CMDs) in adults and asthma in children using published estimates of the underlying disease prevalence in the population to which exposure-related relative risks were applied based on changes in SIT and mould growth, respectively (table 1). Mental health benefit is assumed to persist over 10 years (ie, exponential decay to zero over 10 years).

### Model application: 2030 energy efficiency targets

The model was used to examine the effect of energy efficiency retrofits of the type and order proposed under 2030 GHG mitigation pathways for the English housing sector.^1^ Where dwellings were eligible, the retrofits comprised installing double glazing, insulating cavity and solid walls, adding loft insulation, installing new condensing gas boilers, and adding draught proofing to improve dwelling air tightness in leaky dwellings (air leakage rate ≥7 m^3^/m^2^/h). In addition, non-operational extract fans in the kitchen and bathroom were repaired and window trickle ventilators^ii^ were installed with glazing upgrades.

We examined three scenarios that addressed ventilation alongside the energy efficiency retrofits (table 2). They were:

1. Purpose provided ventilation via extract fans and trickle vents (where not already present) was installed to ensure adequate indoor air quality in line with regulations (Regulation);
2. Purpose provided ventilation was installed (or repaired) only for dwellings that exhibit problems of mould or inadequate ventilation as reported in the EHS (∼1.16 million dwellings—see online supplementary appendix 1; Installer Discretion); and
3. No purpose provided ventilation was added except for repairing broken extract fans and trickle vents for double glazing to reflect the lack of guidance surrounding energy efficiency retrofits (No Added Ventilation).

We assumed instantaneous installation for all retrofits in order to illustrate the effect of changes in exposures and associated health effect with all other unrelated conditions held constant. We also assumed that no changes occurred in the underlying health status of the population over time, an assumption which previous work has shown to have only a minor effect on life table calculations.^41^

**Table 2:** Energy efficiency interventions modelled

Experiment energy efficiency retrofits	Ventilation scenarios		
	Regulation	Installer discretion	No added ventilation
	Number of retrofits installed (1000s)		
Loft insulation	5320	5320	5320
Cavity wall insulation	6560	6560	6560
Solid wall insulation	5700	5700	5700
Double glazing installation	2430	2430	2430
Condensing boiler installation	10 730	10 730	10 730
Gas central heating installation	310	310	310
Draught proofing	3870	3870	3870
Trickle vent and extract fans	15 280	900	0
Extract fan installation only	350	350	0
Extract fan refurbishment	50	50	50
Trickle vent installation only	270	270	0

Note that trickle and extract fans include all new installations, extract fan only already have trickle vents, trickle only already have extract fans.

### Uncertainty analysis

We used Monte Carlo simulation to assess parametric uncertainty in the health impact estimates associated with the determinant of the exposure change (ie, the change in heat loss and air tightness due to each intervention), the exposure–response relationships and the utility weights for each health outcome. We report 95% credible interval estimates based on the 2.5th and 97.5th centiles of results generated from 500 model iterations.^42^ ^43^ See online supplementary appendix 4 for further details.

We also examined the uncertainty of the model due to two important structural assumptions: (1) the length of life lost in those dying of cold-related causes, and (2) the toxicity of particles derived from indoor sources. For cold, assessing chronic health impacts using exposure–response functions based on time series analyses implies that those who are vulnerable to cold-related risks have the same life expectancy as the population average. This is unlikely to be the case; instead it is likely that the people who die of cold-related events are people who have shorter than average life expectancy (see online supplementary appendix 5 for further discussion). To address this, we have examined the effect of assuming that those vulnerable to cold fall into a ‘high-risk’ subgroup of the population with elevated underlying cardiovascular risk. We then examined the shortening of remaining life expectancy in such a high-risk group as a function of (1) its size as a proportion of the total population (if overall cardiovascular deaths remain the same), and (2) the elevation of risk (relative risk) in the high-risk group compared with the remainder of the population. For particle toxicity, the epidemiology is dominated by studies of outdoor air pollution. However, it is unclear whether the same toxicity should be assumed for particles derived from indoor sources, whose concentration may rise if air tightness is increased. To account for this uncertainty, we performed calculations with and without the inclusion of the estimated effect of particles derived from indoor sources.

There is also uncertainty in the use of the mould severity index (MSI) used in the EHS that is derived from a visual inspection of the occurrence and extent of mould on windows, walls and ceilings. The potential uncertainty of the MSI measurement beyond the simple Monte Carlo treatment of the uncertainty in mould exposure is not examined here.

## Results

### Indoor environmental exposure levels

The 2030 energy efficiency interventions resulted in improvements in energy performance, as well as appreciable increases in air tightness. The changes in indoor air pollutant concentrations reflected the ventilation strategy applied under the three different scenarios.^iii^ Table 3 summarises the energy performance, indoor environmental conditions, changes in exposure levels and health impacts.

**Table 3:** Building performance and indoor environment conditions in the English stock for present day (baseline) and cumulative health effect after 50 years for selected exposure-specific diseases under the 2030 energy efficiency retrofit experiment with ventilation scenarios

	Baseline	Experiment ventilation scenarios		
	Intervention stock	Regulation	Installer discretion	No added ventilation
Sample		N		
Dwellings (1000s)		18 990	17 350	17 320
People (1000s)		44 740	41 130	41 060
Building characteristics		Mean (SD*)		
Fabric heat loss (W/K)	294 (167)	219 (120)	213 (115)	213 (116)
Ventilation heat loss (W/K)	75 (45)	70 (42)	51 (35)	50 (33)
Heat system efficiency (%)	76 (12)	88 (11)	89 (10)	89 (10)
Permeability (m^3^/m^2^/h)	16 (5)	11 (5)	11 (5)	11 (5)
Exposure†		Mean (95% credibility intervals)		
Standardised indoor temperature‡ (°C)	17.8 (0.7)	18.1 (18.1, 18)	18.1 (18.1, 18.1)	18.1 (18.1, 18.1)
STS§	0.5 (0.4)	0.5 (0.5, 0.4)	0.7 (0.7, 0.6)	0.7 (0.7, 0.7)
Indoor¶ PM_2.5_ (μg/m^3^)	9.4 (5.4)	4.6 (4.4, 4.2)	10.6 (10.1, 9.6)	11 (10.5, 9.9)
Outdoor PM_2.5_ (μg/m^3^)	6.2 (1.7)	6.8 (6.5, 6.2)	5.9 (5.6, 5.3)	5.8 (5.5, 5.2)
Radon (Bq/m^3^)	22.9 (14.1)	22.4 (20.3, 20.1)	34.2 (30.7, 30)	35 (31.3, 30.7)
Mould (% with MSI >1)	14.9 (7.5)	12.3 (11.6, 11)	18.5 (17.8, 16.2)	18.8 (18.3, 16.5)
Heating energy (MWh/year)	22.9 (10.4)	16.6 (16.4, 16.3)	15.7 (15.6, 15.4)	15.6 (15.5, 15.4)
Health impact**		Total QALYs per 10 000 persons (95% credibility intervals)††		
Cardiovascular (winter)		119 (106, 131)	69 (57, 81)	65 (53, 77)
Heart attack		312 (287, 336)	−232 (−279, −185)	−271 (−319, −223)
Stroke		306 (282, 330)	−258 (−310, −206)	−296 (−349, −242)
Cardiopulmonary		1268 (1169, 1371)	−44 (−83, −6)	−130 (−166, −96)
Lung cancer		233 (209, 258)	−75 (−93, −57)	−97 (−115, −81)
Common mental disorder		2 (2, 4)	3 (3, 4)	3 (3, 4)
Asthma (children)		1 (4, 7)	−1 (−8, −4)	−1 (−9, −5)
Net impact		2241 (2085, 2397)	−539 (−678, −399)	−728 (−864, −592)

*Standard deviation is given for building characteristics as a measure of spread.

†Weighted average values of kitchen (10%), lounge (45%) and bedroom (45%).

‡Average between living room and bedroom temperature when 5°C outdoors.

§STS 1=average exposure level of smoking household.

¶Indoor sources of PM_2.5_ relate to cooking only with an emission rate of 1.6 µg/min.

**Cardiovascular disease is modelled with equal risk across the population and toxicity of indoor and outdoor PM_2.5_ is considered equal and as such the results are likely overestimating the impact—see uncertainty analysis for tests.

††Credibility intervals are derived from Monte Carlo analysis showing using the 5th and 95th centiles from 1000 model iteration results as limits.

MSI, mould severity index; PM_2.5_, particulate matter with a diameter of 2.5 μm or less; STS, secondhand tobacco smoke; QALYs, quality adjusted life years.

Scenario 1 (Regulation), where ventilation systems were added alongside all fabric and heating retrofits, resulted in a 30% reduction in annual heating energy demand, which is aligned with government objectives.^2^ Wintertime temperatures increased by 0.3°C on average (with a SD of ±0.5), while added ventilation reduced indoor sources of pollutants (53% for PM_2.5_, 11% for radon, 13% for STS, 23% for mould), but increased indoor exposure to outdoor-generated PM_2.5_ (4.2%).

The ‘Installer Discretion’ scenario shows that mitigation measures applied due to perceptible conditions of inadequate ventilation or mould growth were insufficient to have wide benefit (in part due to the relatively small number of dwellings exhibiting these conditions, see online supplementary appendix 1). With the added ventilation, heat losses (33%) and heating energy (32%) were greater compared with the ‘Regulation’ scenario along with a modest increase in indoor temperatures. Outdoor sources of PM_2.5_ reduced considerably (-10%), but indoor pollutants experienced sizable increases (8% for PM_2.5_, 34% for radon, 33% for STS and 18% for mould).

Under the ‘No Added Ventilation’ scenario, there were still greater reductions in ventilation heat losses. The average indoor pollutant concentrations were further elevated across the stock compared with scenario 2 (Installer Discretion).

### Health impact of energy efficiency retrofits

**Figure 3:**
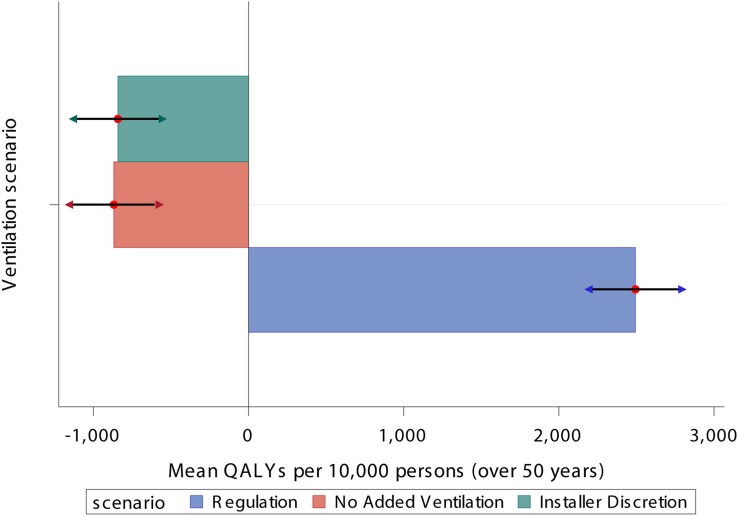
Net mortality and morbidity health effect (quality adjusted life years (QALYs) per 10 000 persons) for all selected exposure-specific diseases after 50 years for the 2030 energy efficiency experiment for different ventilation scenarios (arrows denote 95% credibility intervals). Note: cardiovascular disease is modelled with equal risk across the population and toxicity of indoor and outdoor PM_2.5_ is considered equal and as such the results are likely overestimating the impact—see ‘section, Uncertainty analysis’ for tests (PM_2.5_, particulate matter with a diameter of 2.5 μm or less).

The balance of the overall impact on mortality and morbidity is highly dependent on the assumptions made regarding the level of ventilation to mitigate reduced indoor air quality (table 3; figure 3). Over a follow-up period of 50 years, the net impact of the 2030 energy efficiency interventions under the ‘Regulation’ ventilation scenario resulted in 2241 quality adjusted life years (QALYs) gained per 10 000 persons for the 18.99 million affected dwellings. Selective targeting of ventilation system under the ‘Installer Discretion’ scenario resulted in −539 QALYs per 10 000 persons lost. While no added ventilation had an even greater overall negative impact of −728 QALYs per 10 000 persons lost among the intervention group.

If building regulations were met (scenario 1), the net impact on health is positive primarily because the reduction in exposure to particles of indoor origin is greater than the increase in outdoor-generated particles. Improved indoor temperatures have a net positive effect on cardiovascular disease, though this is dependent on assumptions of the remaining life expectancy of those vulnerable to the effects of cold (see Uncertainty analysis section).

Targeted extract fans and trickle vents in dwellings with a perceptive ventilation problem (scenario 2) offer only moderate modification on the long-term impact on health, a 30% improvement from no additional ventilation (scenario 3). However, despite these interventions, there remained a large number of dwellings that experienced an increase in fabric air tightness.

When no additional ventilation was provided alongside the dwelling energy efficiency retrofits, the increase in indoor sources of air pollutants resulted in a net negative impact on health, despite the reduced ingress of outdoor sources of particulates. Although sensitive to assumptions on the equal toxicity of indoor and outdoor PM_2.5_ (see Uncertainty analysis section), reduced infiltration of outdoor air and increases in exposure to STS, radon and mould risk resulted in a net-negative impact on health.

### Uncertainty analysis

#### Cold-related deaths risk group size

We use here scenario 2 to illustrate the sensitivity of the health impact estimates to changes in the concentration of cardiovascular risk within the population. Reducing the size of the ‘high-risk’ cardiovascular group in the population reduces the scale of the health benefit due to increased winter temperatures, though the overall impact is modest (see table 4). We illustrate this by concentrating the risk across increasingly smaller proportions of the population (from 100% to 0.1%), selected to represent the full range of plausible assumptions. An assumption of 100% of the excess winter cardiovascular deaths being in the high-risk group (ie, the whole population at risk) could result in a considerable overestimate of the change in the burden of winter time cardiovascular disease, while an estimate of 0.1% (ie, only 0.1% of the population are at risk) would effectively remove all of the potential benefit of increased temperatures for population health. Pending further research, it is difficult to estimate the correct level of adjustment. However, the impact is almost certain to be appreciably less than that implied by using time series coefficients applied without any correction.

**Table 4:** Cumulative health effect after 50 years for varying high-risk excess winter cardiovascular group size under the 2030 energy efficiency retrofit experiment for scenario 2 ‘installer discretion’

	Experiment ventilation scenario 2: ‘Installer Discretion’			
	Size of ‘high-risk’ group*			
	100%†	10%	1%	0.1%
Net QALYs	Mean per 10 000 persons (95% credibility intervals)			
Cardiovascular (winter)	68.8 (56.8, 80.7)	34.1 (28.1, 40)	14.5 (12, 17)	4.8 (4, 5.7)
Heart attack	−232.1 (−279.1, −185.2)	−232.6 (−277.1, −188.1)	−232.7 (−276, −189.5)	−232.2 (−275.3, −189)
Stroke	−257.6 (−309.7, −205.5)	−257.2 (−307, −207.4)	−256.3 (−304.4, −208.2)	−257.3 (−305.5, −209.1)
Cardiopulmonary	−44.3 (−83.4, −5.6)	−46.6 (−85.6, −8.1)	−47.4 (−86.7, −8.8)	−44.2 (−83.4, −5.4)
Lung cancer	−74.9 (−92.9, −57.4)	−74.3 (−91.9, −57.2)	−75 (−92.9, −57.7)	−74.9 (−92.9, −57.5)
Common mental disorder	2.7 (2.8, 4.1)	2.7 (2.8, 4)	2.8 (2.8, 4.1)	2.7 (2.8, 4)
Asthma (children)	−1.3 (−8.4, −4.3)	−1.3 (−8.4, −4.4)	−1.3 (−8.4, −4.3)	−1.3 (−8.2, −4.2)
Net impact	−538.6 (−677.9, −399.3)	−575.2 (−706.5, −443.9)	−595.5 (−724.2, −466.7)	−602.2 (−729.6, −474.8)

*Proportion of the population in the group assumed to be at high risk for cardiovascular events.

†100% equivalent to whole population equally at risk.

QALYs, quality adjusted life years.

#### Toxicity of indoor particulate matter

There is uncertainty about the relative toxicity of particles generated from indoor sources compared with those from outdoor sources. Some evidence suggests these might be as toxic or perhaps even more toxic as particulate matter (PM) derived from outdoor sources.^35^ ^36^ Analysis in which indoor-generated PM_2.5_ was assumed to have no adverse effect on health had a significant impact on the results (see table 5), reducing the overall net health impact by around 78% compared with the base case results (which assumed equal toxicity to outdoor particulates). Though the effect may be uncertain, there is very likely to be some impact from indoor sources and we would stress the need for more empirical studies that measure and assess the toxicity of indoor PM_2.5_, and the balance of indoor and outdoor particles on health.

**Table 5:** Cumulative health effect after 50 years for indoor PM_2.5_ toxicity equal to outdoor sources and with no effect of indoor PM_2.5_ under the 2030 energy efficiency retrofit experiment for scenario 2 ‘installer discretion’

	Experiment ventilation scenario 2	
	Indoor particulate matter toxicity	
	Equal to outdoor	No effect
Net QALYs	Mean per 10 000 persons (95% credibility intervals)	
Cardiovascular (winter)	68.8 (56.8, 80.7)	81.6 (69.8, 93.4)
Heart attack	−232.1 (−279.1, −185.2)	−186 (−225, −147)
Stroke	−257.6 (−309.7, −205.5)	−212.1 (−255.1, −169)
Cardiopulmonary	−44.3 (−83.4, −5.6)	200.8 (170.5, 233.5)
Lung cancer	−74.9 (−92.9, −57.4)	−47 (−59.8, −34.5)
Common mental disorder	2.7 (2.8, 4.1)	2.8 (2.9, 4.1)
Asthma (children)	−1.3 (−8.4, −4.3)	−1.3 (−8.1, −4.2)
Net impact	−538.6 (−677.9, −399.3)	−161.2 (−240.3, −82)

PM_2.5_, particulate matter with a diameter of 2.5 μm or less; QALYs, quality adjusted life years.

## Discussion

This modelling work shows that predicted changes in indoor environmental exposures following housing energy efficiency interventions of the type being proposed by the UK Government may have an appreciable impact on health. This approach can be applied to different country settings but with regard to existing conditions, and information on the housing stock and households therein.

There is an expectation that retrofits that seek to reduce space heating energy demand will increase indoor temperatures,^12^ but such interventions will also affect the dwelling air tightness and its ventilation. Although indicative, our modelling suggests that reducing fabric heat loss and increasing air tightness may reduce exposure to outdoor pollutants and raise indoor temperatures. However, without added ventilation, indoor concentrations are increased with associated adverse health impacts which are greater than those associated with indoor temperatures, leading to an overall negative impact on health. As demonstrated, this conclusion is sensitive to assumptions made about the toxicity of particles from indoor sources, an area where further research is urgently needed.

In the various scenarios, for purposes of illustration, we assumed an instantaneous installation and a lagged health impact associated with step changes in some exposures. However, the reality will be that these interventions and potential impacts will be realised over a longer period of time. Under the UK's mitigation targets, virtually all English dwellings will need retrofitting by 2030 (ie, 20 million over 15 years or 3650 per day). Putting in place effective measures to address ventilation now can have long-term health effects for both existing and future households.

Although associations between indoor temperatures and mental well-being have been reported,^38^ it is unclear how long the benefit to mental well-being would persist following improved temperatures. Given the high prevalence of CMD in the population, any small shift can be highly influential on the results. While there is very likely to be benefit that accrues beyond a single year and maybe a seasonal effect for a period afterwards, the long-term benefit will likely be affected by the risk of reoccurring episodes of mental health driven by factors other than thermal environment.

The underlying assumptions regarding housing air tightness and occupant ventilation practices (eg, window opening behaviour) are both extremely important. The EHS shows that 71% of homes have no extract fans (or working extract fans); in other words, these homes are naturally ventilated and thus, the exposure to indoor-generated pollutants will be highly determined by the air tightness of the dwelling and the practices of the occupants. Our model has examined the uncertainty of these practices on our estimates and therefore, provides a reasonable spread on the likely true impact.^43^ From our scenarios, we found that added ventilation accompanying efficiency retrofits mitigated the health risk associated with increased air tightness (scenario 1), but that this mitigation must be applied beyond ‘problem homes’ (scenario 2), only the widespread installation of ventilation systems results in a net benefit to health (scenario 1), and providing no additional ventilation poses a potential risk to health (scenario 3).

The provision of added ventilation to offset potential increases in indoor concentrations of pollutants following fabric energy retrofits is an important issue for public health. While the spirit of the building regulations suggests that adequate ventilation should be provided following changes to a dwelling, there is no explicit guidance for installers on what and when to install such systems. The Housing Health and Safety Rating System provides an ‘after-the-fact’ route through which remediation of poor indoor air quality could be addressed, but it is both unlikely and undesirable to rely on this system to address issues that could otherwise be avoided. Clearly assumptions on how a household ventilates their dwelling will have an important impact on creating a healthy indoor environment. Dwellings with higher ventilation rates have been shown to have reduced health burdens,^10^ ^44^ though the association with air change rates and specific diseases can be equivocal.^45^ Occupant ventilation practices have also been shown to be counter-productive to creating a healthy indoor environment. A study of Dutch households showed that many neglect the annual maintenance required to ensure that ventilation system operation is not compromised.^46^ Education around ventilation will be essential to minimise exposure to indoor pollutants following retrofits. Our work highlights that the potential health impacts following efficiency retrofits are not necessarily positive and that there may be risk trade-offs that will depend on the retrofit installation regulatory framework. Having stronger regulation around energy efficiency retrofits and ventilation will help to realise multiple benefits (eg, energy savings and health).

## Strengths and limitations

Modelling studies provide a method of examining complex problems by drawing together data from a range of sources in order to explore the potential impact of interventions on population health. While quantifying the potential health impact of policy options is preferable over qualitative assessment, doing so is subject to several difficulties, primarily the availability of evidence^47^ and the potential to add scientific credibility to uncertain predictions.^48^ The modelling also involves many uncertainties. For instance, the limited set of observed data on how such retrofits affect indoor air quality remains an impediment, with only a few studies looking at the determinants of indoor air quality (eg, infiltration).^5^ There is a paucity of evidence relating to some of the most important health outcomes—especially in relation to cold.^49^ In the overall balance of health calculations, morbidity impacts are potentially larger than those of mortality, for example, the effect of improved temperatures on CMD,^5^ but the evidence is still uncertain, and this gap in the research evidence should be addressed.

The modelling results are presented as QALYs; however, it is clear that these changes in disease outcomes would have an impact on health and social care services beyond these utility estimates. As the average age of the UK population increases so too does the demand on health services. Preventative actions, such as improving energy and ventilation performance, may help to mitigate some of this demand.

The exposure modelling in this experiment concentrated on indoor conditions. The experiment did not alter outdoor pollutant concentrations related to proposed energy supply decarbonisation,^1^ which may reduce outdoor levels of particulate matter in the future.^50^ This would further tip the balance towards installing mitigating ventilation systems so as to dilute ‘stale’ indoor air. Refining the model to include assumptions on energy systems and transport could further improve the estimates of the potential health impact associated with UK's GHG abatement measures.

## Conclusions and policy implications

On balance, if properly implemented, actions to mitigate climate change through energy efficiency in housing can have benefits to health by reducing exposure to cold and outdoor air pollutants. They will also offer indirect health benefits by providing more resilience to protect indoor thermal conditions during extreme cold and heat events. Modelling studies of the type presented here are needed to ensure housing policies are developed in ways that capitalise on this potential for improving health. Such studies, however, should be used with acknowledgment of their uncertainty and limitations, and do not supplant the need for well-designed empirical studies that can validate models and offer policymakers more evidence, and provide greater confidence around policy impact.

We have shown that, unless specific remediation is used, reducing the ventilation of dwellings will improve energy efficiency at the expense of increased exposure to indoor air pollutants and risk to health. However, an important conclusion of this work is that, with careful attention to retrofit installation and ventilation practices, these potential negative impacts can be removed.

The policy agenda and evidence base on the health impact of home energy efficiency is still evolving. Guidance for installers regarding adequate levels of ventilation to protect health is now needed before the large-scale introduction of energy efficiency measures into the housing stock.

## Footnotes

i The standardised internal temperature (SIT) is derived from an empirical study of 1600 English dwellings with half-hourly temperature measurements for a period of 2–4 weeks over the winter period of 2001/2002 and 2002/2003. The SIT is derived from regression models of indoor on outdoor temperature for each dwelling. The models are used to derive a predicted indoor temperature at 5°C outdoor temperature.^12^

ii A small purpose provided opening in a window or building envelope that facilitates ventilation in spaces when large openings (windows and doors) are closed and fans are turned off.

iii The modelled estimates for the baseline housing stock energy performance and indoor exposures were compared against observed national and sample stock distributions to check the accuracy of the outputs (see online supplementary appendix 1).
