# Correction

**DOI:** 10.1136/bmjopen-2014-007298corr1

**Published:** 2017-04-22

Hamilton I, Milner J, Chalabi Z*, et al.* Health effects of home energy efficiency interventions in England: a modelling study. *BMJ Open* 2015;5:e007298. doi: 10.1136/bmjopen-2014-007298

Some text was missed out of the Acknowledgements in the original paper. The full Acknowledgement statement should read:

**Acknowledgements** The following persons were involved in the initial 2009 ‘Health impact of energy efficiency’ DECC funded project—LSHTM: Hutchinson E; Sheffield Hallam University: Wilson I, Green G, Gilbertson J, Stafford B; Warwick University: Ormandy D; Ulster University: Liddell C, Morris C; UCL: Ian Ridley. Ian Ridley was also involved in the subsequent 2012 funded work, developing core parts of the pollutant module.

